# Adverse drug reaction reporting and development of pharmacovigilance systems in Bosnia and Herzegovina, Croatia, Serbia, and Montenegro: a retrospective pharmacoepidemiological study

**DOI:** 10.3325/cmj.2018.59.124

**Published:** 2018-06

**Authors:** Una Glamočlija, Biljana Tubić, Martin Kondža, Aleksandar Zolak, Nataša Grubiša

**Affiliations:** 1 School of Medicine, University of Mostar, Mostar, Bosnia and Herzegovina; 2 Agency for Medicines and Medical Devices, Banja Luka, Bosnia and Herzegovina; 3 School of Pharmacy, University of Mostar, Mostar, Bosnia and Herzegovina

## Abstract

**Aim:**

To compare individual case safety reports (ICSR) rates and characteristics between Croatia, Serbia, Montenegro, and Bosnia and Herzegovina (B&H).

**Methods:**

This retrospective pharmacoepidemiological study used the data from ICSR received by the Agency for Medicines and Medical Devices in B&H in 2011-2016. The number, characteristics, and sources of reports, suspected drugs, and patient characteristics were analyzed. The results were compared with the publicly available data from Croatia, Serbia, and Montenegro.

**Results:**

The number of reported adverse drug reactions per one million of inhabitants was lowest in B&H and highest in Croatia. There were significant differences in reporter characteristics, sources of reports, and the percentage of missing data in ICSR, while the Anatomical Therapeutic Chemical product classes, patient’s sex, and adverse drug reaction System Organ Classes were similar.

**Conclusion:**

Despite the historical and geographical vicinity of B&H and its neighboring countries, there were significant differences in indicators of pharmacovigilance development.

Preclinical and clinical investigations of a medicinal product cannot provide complete information on its safety. Therefore, post-marketing evaluation is needed to collect data on adverse effects and contribute to the safer use of medicinal product. To answer the questions related to adverse drug reactions (ADR), every country has to have a well-organized pharmacovigilance system (1). The development of national pharmacovigilance systems can be evaluated by the number and quality of individual case safety reports (ICSR) and report sources. Other characteristics listed in ICSR, such as Anatomical Therapeutic Chemical (ATC) classes of products, ADRs, and patient characteristics, may be influenced by different factors (physicians' prescribing habits or patients' medicine-taking habits) and can be used for additional analysis of pharmacovigilance systems.

Bosnia and Herzegovina (B&H) and its neighboring countries – Croatia, Serbia, and Montenegro were part of Yugoslavia until 1991. Yugoslavia had a well-organized pharmacovigilance system and participated in the World Health Organization (WHO) Programme for International Drug Monitoring since 1974 (2). Although almost 30 years have passed from the breakup of Yugoslavia, the four countries still have similar pharmacovigilance regulations (Table 1). While regulatory bodies responsible for drug safety in Croatia (3), Serbia (4), and Montenegro (5) publish yearly pharmacovigilance reports on their web pages, no reports from B&H have been available. This study for the first time presents and analyzes the reports from B&H. Also, there has been no comparison of national pharmacovigilance systems in Croatia, Serbia, Montenegro, and B&H. Such comparison may serve as a valuable model showing how different practice and outcomes of pharmacovigilance in countries with similar pharmacovigilance regulations may lead to different development levels of national pharmacovigilance systems. The aim of this study was to compare ICSR rates and ICSR characteristics in Croatia, Serbia, Montenegro, and B&H, as indicators of the development level of national pharmacovigilance systems.

**Table 1:** Characteristics of pharmacovigilance systems in Bosnia and Herzegovina (B&H), Croatia, Serbia, and Montenegro*

Characteristic	Country			
	B&H	Croatia	Serbia	Montenegro
EU member	Potential candidate for EU membership	Yes	Potential candidate for EU membership	Potential candidate for EU membership
WHO Programme for International Drug Monitoring member	No, associated member	Yes	Yes	Yes
Valid regulations	Rulebook on the Manner of Reporting, Collecting and Following Adverse Effects of The Medicine (Official Gazette in B&H, No. 58/2012) Medicines and Medical Devices Act (Official Gazette in B&H, No. 58/2008)	Medicinal Product Act (National Gazette No. 76/2013) Ordinance on Pharmacovigilance (Official Gazette No. 83/2013) All regulations harmonized with current EU regulations	Medicines and Medical Devices Act (The Official Gazette of the RS, 30/2010) Rulebook on the Method of Reporting, Collecting and Monitoring Adverse Reactions to Medicines (The Official Gazette of the Republic of Serbia, 64/2011)	Medicines Act (Official Gazette of Montenegro, No. 56/2011) Rulebook on the Manner of Collecting of Data and Reporting and Monitoring Adverse Reactions to Medicines for Use in Human Medicine (Official Gazette of Montenegro, No. 46/2014)
Regulatory body responsible for pharmacovigilance	ALMBIH (27)	HALMED (7)	ALIMS (12)	CALIMS (28)
Adverse drug reaction reporting	Obligatory for drug producers, health care institutions, and professionals. Reports are sent to ALMBIH.	Mandatory for health care professionals, manufacturers, MAHs, holders of authorization for parallel import, and wholesalers. Reports are sent to HALMED. Patient can report to health care professional or to HALMED directly.	Mandatory for health care professionals and patients/ medical product users. Reports are sent to the regional pharmacovigilance center or the ALIMS.	Obligatory for MAH, health and veterinary institutions, and professionals, sponsors of clinical trials and legal entities involved in a drug market. Reports are sent to CALIMS.

*ALIMS – Medicines and Medical Devices Agency of Serbia; ALMBIH – Agency for Medicines and Medical Devices in B&H; CALIMS – Agency for Medicines and Medical Devices of Montenegro; EU – European Union; HALMED – Agency for Medicinal Products and Medical Devices of Croatia; MAH – marketing authorization holder, WHO – World Health Organization.

## Material and methods

This retrospective pharmacoepidemiological study analyzed ICSR received by the Agency for Medicines and Medical Devices in B&H (ALMBIH) in the period 2011-2016. The results were compared with the publicly available results from Croatia, Serbia, and Montenegro (3-5). We also analyzed the activities of ALMBIH from their own data and the activities of the relevant institutions in Croatia, Serbia, and Montenegro from their publicly available data (3-5). The study was performed by ALMBIH between February 2017 and January 2018.

### Material

ICSR received by the ALMBIH by mail, email, fax, or through online reporting system for marketing authorization holders (MAH) were used as data source for B&H. For Croatia, Serbia, and Montenegro, data were obtained from publicly available yearly reports. For Croatia and Montenegro, the reports were available for the period 2011-2016. For Serbia, the reports were available for the period 2011-2015 and did not contain all relevant data. Additional data could not be obtained from Medicines and Medical Devices Agency of Serbia (ALIMS) directly upon request. Thus, for comparison of the development level of pharmacovigilance systems, available data from Serbia were used. Data on B&H population were obtained from the Agency for Statistics of B&H (6).

### Methods

ICSR received by ALMBIH were entered into Microsoft Excel^TM^ tables (Microsoft Corporation, Redmond, WA, USA). The number, characteristics, and sources of ICSR, suspected drugs, ADRs, and patient characteristics were analyzed quantitatively. The first level of ATC classification was used to characterize suspected drugs in ICSR. ADRs were coded according to the Medical Dictionary for Regulatory Activities System Organ Class (SOC) classification. One ICSR represented one report for a specific patient and can contain more than one ADR and more than one suspected drug. Therefore, the number of ADRs and suspected drugs was higher than the total number of ICSR. Previously published data from Croatia (3), Serbia (4), and Montenegro (5) were entered into Microsoft Excel^TM^ tables, analyzed, and presented in the form of charts and tables.

### Statistical analysis

The normality of data was evaluated by Shapiro-Wilks test. The number of ICSR per one million of inhabitants, percentage of pharmacists, physicians, health care professionals, MAH, and patient reporters and percentage of female patients were normally distributed. The significance of the relationship between analyzed parameters was assessed using type I analysis of variance (ANOVA) test. Since a part of data for Serbia was missing, Games-Howell *post-hoc* test was used for comparison. *P* < 0.05 was considered statistically significant. Statistical analysis was performed using IBM SPSS Statistics for Windows, version 24.0 (IBM Corp., Armonk, NY, USA, licensed to the corresponding author).

## Results

### ICSR reporting

**Figure 1:**
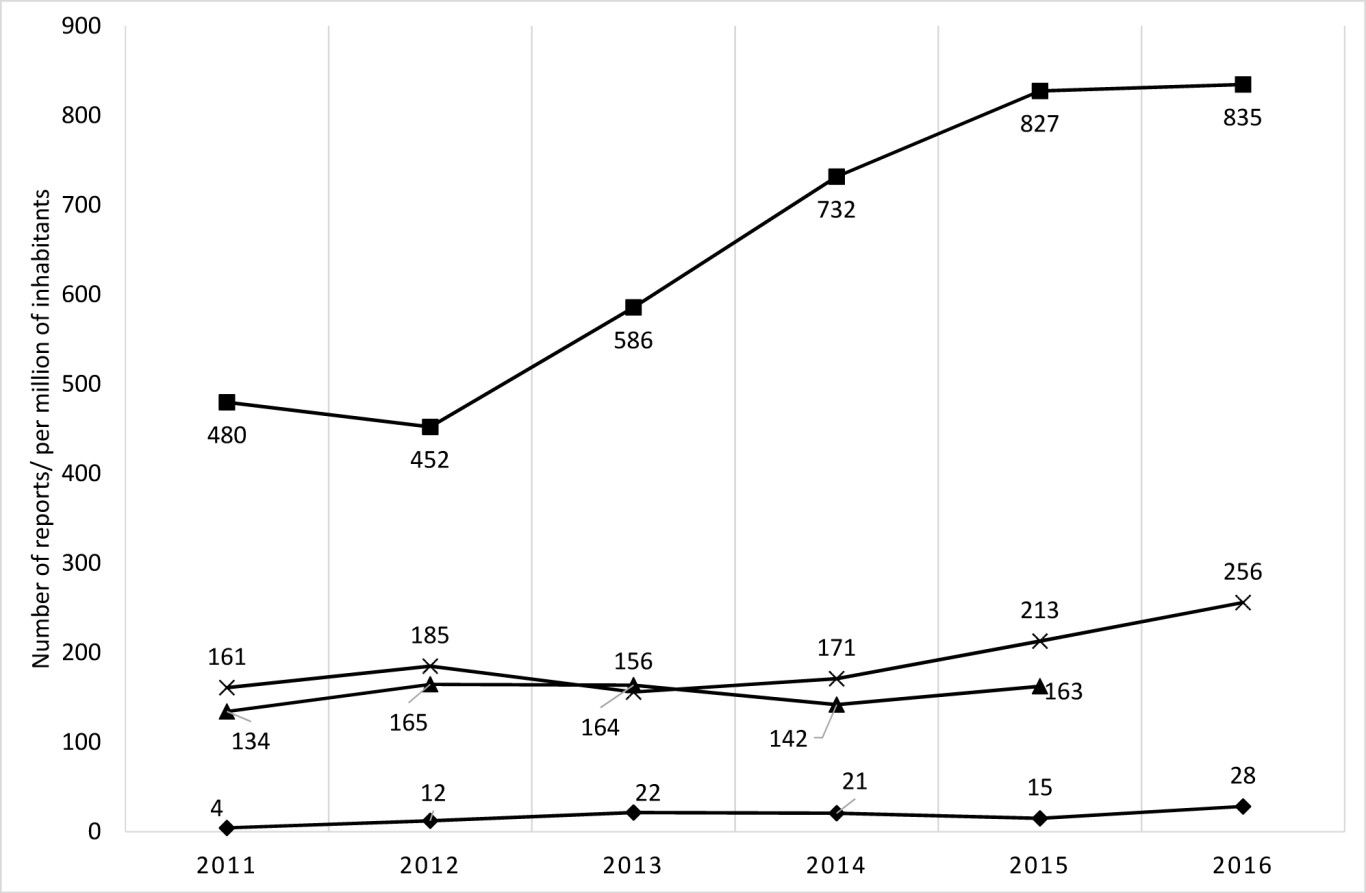
Differences in the number of individual case safety reports per one million of inhabitants between Bosnia and Herzegovina (rhomb) and neighboring Croatia (square), Serbia (triangle), and Montenegro (cross).

The number of ICSR was lowest in B&H and highest in Croatia. Serbia and Montenegro had similar number of reports, which was lower than in Croatia and higher than in B&H (Figure 1).

There was no significant difference in the percentage of MAH reporters among the four countries (Table 2). Patient reporting was present only in Croatia (Serbia had a very small percentage of patient reporters). The percentage of patient reporters from Croatia was constantly growing, and peaked in 2016, when 10% of all reports were patients’ reports (Table 2). There was no significant difference in the percentage of health care professionals among the total number of reporters (Table 2). Among health care professionals, physicians were the most frequent reporters in all four countries. Croatia had the lowest percentage of physician reporters (Croatia versus B&H, Serbia, and Montenegro; *P*=0.027, *P*=0.026, and *P*=0.003, respectively), while no significant difference was found between B&H, Serbia, and Montenegro (Table 2).

**Table 2:** Sources of reporting in Bosnia and Herzegovina (B&H), Croatia, Montenegro, and Serbia*

	Sources of reporting per year (%)					
Country	2011	2012	2013	2014	2015	2016
**B&H**						
MAH	27	44	59	30	43	53
Patients	0	0	0	0	0	0
Unknown	20	2	1	1	2	1
Healthcare professionals	53	54	40	68	55	46
physician	60	66	92	93	83	96
pharmacist	20	12	7	4	9	3
other	20	22	1	3	8	1
**Croatia**						
MAH	33	34	29	23	35	26
Patients	1	2	6	6	7	10
Healthcare professionals	66	63	65	71	58	64
physician	65	55	49	57	55	53
pharmacist	24	32	36	40	40	40
other	10	13	15	3	6	7
**Serbia**						
MAH	64	60	41	39	51	NA
Patients	0	0	1	1	2	NA
Healthcare professionals	36	40	58	61	47	NA
physician	NA	NA	69	73	68	NA
pharmacist	NA	NA	29	26	30	NA
other	NA	NA	2	1	2	NA
**Montenegro**						
MAH	39	40	42	42	28	65
Patients	0	0	0	0	0	0
Healthcare professionals	61	60	58	58	72	35
physician	98	96	97	81	67	87
pharmacist	2	2	1	18	30	13
other	0	2	2	1	4	0

*MAH – marketing authorization holder.

Percentage of pharmacist reporters in Croatia was significantly higher than in B&H and Montenegro (*P* < 0.001 and *P* = 0.009, respectively) (Table 2). Data on the percentage of pharmacist reporters from Serbia were available only for 2013-2015 period, and no significant difference was found in comparison with Croatia and Montenegro; still, it was significantly higher than in B&H (*P* = 0.004) (Table 2). There was no significant difference in the number of pharmacist reporters between B&H and Montenegro.

### ATC classes of products, patient characteristics, and ADRs in ICSR

**Figure 2:**
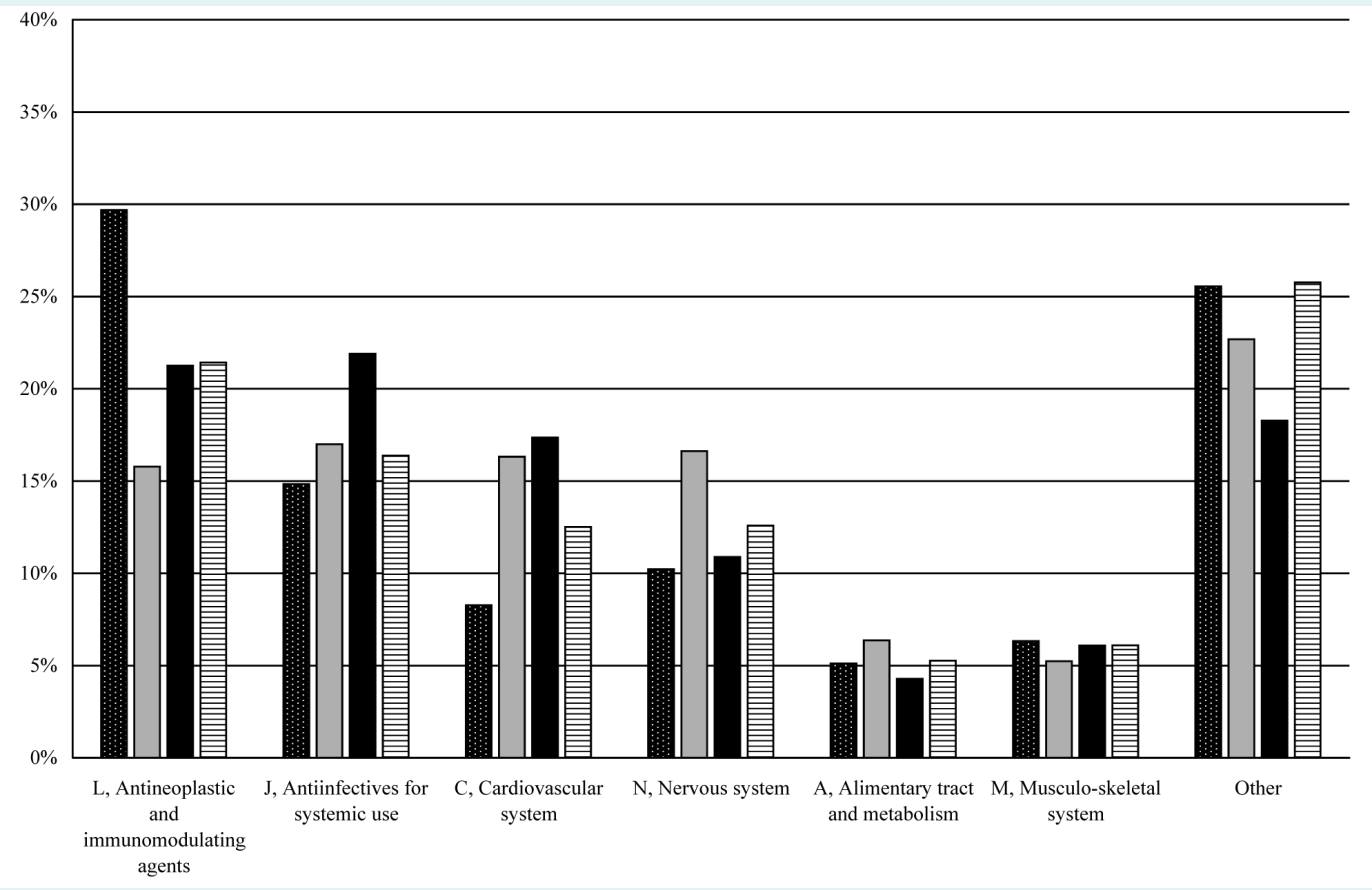
Anatomical Therapeutic Chemical classes for suspected drugs in reports from Bosnia and Herzegovina (B&H, black with dots), Croatia (gray), Montenegro (black) and Serbia (white with horizontal lines). Cumulative data for 2011-2016 for B&H, Croatia, and Montenegro and 2011-2015 for Serbia.

**Figure 3:**
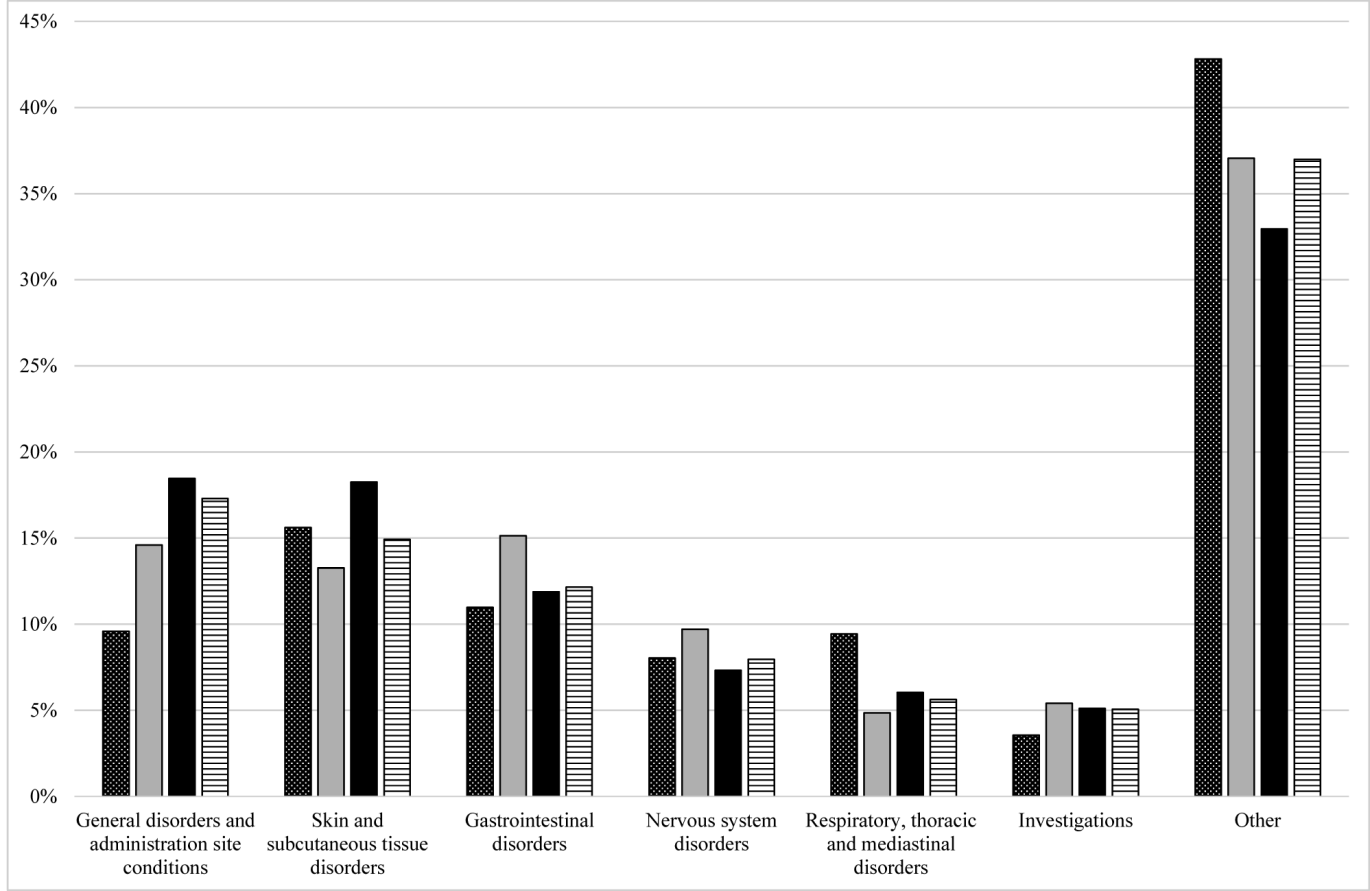
System Organ Class of adverse drug reactions in individual case safety reports from Bosnia and Herzegovina (black with dots), Croatia (gray), and Montenegro (black) cumulatively for 2011-2016 and Serbia (white with horizontal lines) for 2014 and 2015.

ATC classes were similar in all countries (Figure 2). Most patients were women, except in B&H in 2015, when there were 49% male patients, 43% female patients, and 8% patients of unknown sex. There was no significant difference among the countries in the percentage of female patients. In 2011 and 2012, a high percentage of ICSR from B&H contained no information on patents’ sex and age. After 2012, this percentage decreased (Table 3). In Croatia, Serbia, and Montenegro, patients' age and sex were frequently reported in the entire analyzed period (Table 3). SOC classes were similar in all countries (Figure 3).

**Table 3:** Individual case safety reports (ICSR) containing no data on sex and age of patients from Bosnia and Herzegovina (B&H), Croatia, Serbia, and Montenegro between 2011 and 2016

Unknown data on	No. (%) of ICSR per year					
	2011	2012	2013	2014	2015	2016
**Sex**						
B&H	7 (47.0)	16 (39.0)	7 (9.0)	3 (4.0)	4 (8.0)	4 (4.0)
Croatia	28 (1.0)	52 (3.0)	84 (3.0)	134 (4.0)	189 (5.0)	66 (2.0)
Serbia	17 (2.0)	31 (3.0)	0 (0.0)	81 (8.0)	47 (5.0)	NA
Montenegro	0 (0.0)	0 (0.0)	0 (0.0)	1 (1.0)	2 (2.0)	6 (4.0)
**Age**						
B&H	7 (47.0)	16 (39.0)	5 (7.0)	5 (7.0)	1 (2.0)	6 (6.0)
Croatia	178 (9.0)	269 (14.0)	321 (13.0)	409 (13.0)	453 (13.0)	398 (11.0)
Serbia	0 (0.0)	99 (9.0)	0 (0.0)	160 (16.0)	177 (16.0)	NA
Montenegro	2 (3.0)	6 (5.0)	5 (5.0)	5 (5.0)	5 (4.0)	3 (2.0)

## Discussion

There were significant differences between the four countries in the ICSR number, sources, and percentages of missing data in ICSR. The data for dominant ATC classes of products, patients' sex, and ADR SOC classifications were similar.

The number of ICSR in a country directly indicates the pharmacovigilance system development. According to WHO, each national pharmacovigilance center should send at least 200 ICSR per million inhabitants yearly (7). B&H had a very low number of reports during the analyzed period. The main reasons might be lack of dedicated employees in ALMBIH and lack of relevant education for reporters. In the analyzed period in B&H, no larger campaigns or pharmacovigilance educations were carried out by ALMBIH and other relevant institutions. Reporting forms and educational materials were available on the ALMBIH web page. No patient reporting was described in relevant regulations, and no innovative reporting forms for patients or health care professionals were developed. Online or other reporting solutions were not developed. Amrain and Bečić (8) found that only 15.4% of 870 surveyed health care professionals in B&H had previously reported ADRs due to reluctance to admit harm to patient, lack of time, and a complicated reporting form, although 92.6% of respondents thought that pharmacovigilance was important. Another survey found that pharmacists and physicians from B&H thought that pharmacovigilance education needs to be improved (9).

Croatia, on the other hand, had the highest number of ICSR, which was constantly growing during the analyzed period. Croatia has more than 40-year-long pharmacovigilance tradition, as the National Pharmacovigilance Center of Yugoslavia was located in Zagreb (10). The Agency for Medicinal Products and Medical Devices of Croatia (HALMED) was among the first three national agencies for medicines in the European Union using mobile application for ADR reporting. HALMED has special forms for patient reporting (11,12) and it included patient reporting in the relevant regulations (Table 1).

Serbia and Montenegro had similar number of reports, which was lower than in Croatia and higher than in B&H. In Montenegro, the Agency for Medicines and Medical Devices of Montenegro (CALIMS) plays a very important role in the pharmacovigilance promotion and education. A reporting system is constantly being upgraded, with the main focus on online options. Online forms are well-developed and have many mandatory fields. CALIMS developed projects and collaborations for pharmacovigilance system improvement (13). In Serbia, because of the lack of yearly pharmacovigilance reports, the last having been published for 2015, and no relevant data available on the ALIMS web pages, the pharmacovigilance activities could not be reliably identified. ALIMS provided special forms for patient reporting (14) and included patient reporting in the relevant regulations (Table 1).

Types of reporters are among the main indicators of the awareness of ADRs reporting importance. Considerable level of patient reporting was only present in Croatia. This might be explained by HALMED activities in pharmacovigilance education of patients. Croatia was the first country in the world to launch a WHO online reporting system for patients in 2012 (15). Pharmacists also play a significant role in this system since they directly contact the patients using over-the-counter drugs and products. Montenegro is an example of how specific activities can change the pharmacovigilance system. In 2014, CALIMS strengthened collaboration with pharmacies and Pharmaceutical Chamber of Montenegro, after which the number of pharmacist reporters increased (Table 2) (13,16).

Quality of reports can be evaluated through the percentage of unknown data. High percentage of ICSR with unknown sex and age data from B&H for 2011 and 2012 indicates the low quality of reports. The decrease in the percentage of incomplete reports after 2012 could be attributed to education of health care professionals performed through ALMBIH website and lectures at relevant conferences.

There is a number of studies on the difference between pharmacovigilance regulations and development between countries (17-22), but to our knowledge, this is the first study to evaluate how countries that were part of the same country can develop pharmacovigilance systems with various development levels. Limited financial resources slow down pharmacovigilance development, and countries with limited resources can have less developed pharmacovigilance systems (17,23). Moreover, projects aimed at faster development of pharmacovigilance systems generally give direct results (24).

A limitation of our study is a lack of some relevant data (for Serbia for 2016 and partly for previous years). Moreover, the analyzed period was short and the number of analyzed countries small. To assess the factors affecting the pharmacovigilance system development we should analyze a larger number of countries during a longer period.

Since pharmacovigilance plays a crucial role in safer drugs use, all countries should put in effort to develop well organized systems, producing enough relevant data to optimize risk-benefit analysis in therapy. Some of the activities for countries with less developed pharmacovigilance systems should be continuous education of health care professionals, leading to their active participation in pharmacovigilance; more intensive pharmacists' involvement in ADR reporting; regulation and improvement of patients’ reporting; implementation of online and other adequate tools and ADR reporting systems accessible to all potential reporters; and cooperation and exchange of information between countries.

## References

[1] Sharrar RG, Dieck GS (2013). Monitoring product safety in the postmarketing environment.. Ther Adv Drug Saf.

[2] Stephens M. The dawn of drug safety. 2nd ed. Winchester: George Mann Publications; 2012.

[3] Adverse Drug Reactions Reports. HALMED [in Croatian]. Zagreb: Agency for Medicinal Products and Medical Devices of Croatia. Available from: http://www.halmed.hr/en/Farmakovigilancija/Izvjesca-o-nuspojavama/. Accessed: March 22, 2017.

[4] Annual report – ALIMS [in Serbian]. Belgrade: Medicines and Medical Devices Agency of Serbia. Available from: http://www.alims.gov.rs/latin/farmakovigilanca/godisnji-izvestaji/ Accessed: April 4, 2017.

[5] Agency for Medicines and Medical Devices of Montenegro – CALIMS. Podgorica: Agency for Medicines and Medical Devices of Montenegro. Available from: https://www.calims.me/Portal/faces/dinamickeStrane?paramPut=About+Agency++%253E++Activities&paramS=5&_afrWindowMode=0&_afrLoop=29581508845650472&paramRender=2&_adf.ctrl-state=13kn9g0ozo_4*.* Accessed: April 4, 2017.

[6] Bosnia and Herzegovina. Agency for Statistics. Available from: http://www.bhas.ba/index.php?lang=en. Accessed: April 4, 2017.

[7] Rosli R, Ming LC, Abd Aziz N, Manan MM (2016). A retrospective analysis of spontaneous adverse drug reactions reports relating to paediatric patients.. PLoS One.

[8] Amrain M, Bečić F (2014). Knowledge, perception, practices and barriers of healthcare professionals in Bosnia and Herzegovina towards adverse drug reaction reporting and pharmacovigilance.. Journal of Health Sciences.

[9] Catic T, Begović B (2016). The attitudes of pharmacists and physicians in Bosnia and Herzegovina towards adverse drug reaction reporting.. J Health Sci.

[10] HALMED Pharmacovigilance [in Croatian]. Zagreb: Agency for Medicinal Products and Medical Devices of Croatia. Available from: http://www.halmed.hr/Farmakovigilancija/40-godina-spontanog-prijavljivanja-nuspojava-u-Hrvatskoj/Povijesni-prikaz-pracenja-sigurnosti-primjene-lijekova-u-Hrvatskoj/*.* Accessed: June 2, 2018.

[11] Ordinance on Pharmacovigilance [in Croatian]. Croatian Official Gazette, 83/2013 (2013).

[12] Primary report [in Croatian]. HALMED Patient Reporting. Zagreb: Agency for Medicinal Products and Medical Devices of Croatia. Available from: https://primaryreporting.who-umc.org/Reporting/Reporter?OrganizationID=HR*.* Accessed: March 22, 2017.

[13] Report of the on the Agency for Medicines and Medical Devices of Montenegro on reporting of drug side effects for 2016 [in Montenegrin]. Podgorica: Agency for Medicines and Medical Devices of Montenegro; 2017.

[14] Rulebook on the method of reporting, collecting and monitoring adverse reactions to medicines [in Serbian]. The Official Gazette of the Republic of Serbia, 64/2011 (2011).

[15] Report of the Agency for Medicinal Products and Medical Devices of Croatia (HALMED) on the reporting of suspicious drugs side effects in the Republic of Croatia for 2016 [in Croatian]. Zagreb: Agency for Medicinal Products and Medical Devices of Croatia; 2017.

[16] Report of the on the Agency for Medicines and Medical Devices of Montenegro on reports of drugs side effects for 2014 [in Montenegrin]. Podgorica: Agency for Medicines and Medical Devices of Montenegro; 2015.

[17] Elshafie S, Zaghloul I, Roberti AM (2017). Pharmacovigilance in developing countries (part I): importance and challenges.. Int J Clin Pharm.

[18] Olivera ME, Uema SA, Romańuk CB, Caffaratti M, Mastroianni PC, Varallo FR (2014). Regulatory issues on pharmacovigilance in Latin American countries.. Pharmaceuticals, Policy and Law..

[19] Ampadu HH, Hoekman J, de Bruin ML, Pal SN, Olsson S, Sartori D (2016). Adverse drug reaction reporting in Africa and a comparison of individual case safety report characteristics between Africa and the rest of the world: analyses of spontaneous reports in VigiBase®.. Drug Saf.

[20] Biswas P (2013). Pharmacovigilance in Asia.. J Pharmacol Pharmacother.

[21] Wilbur K (2013). Pharmacovigilance in the Middle East: a survey of 13 Arabic-speaking countries.. Drug Saf.

[22] Suwankesawong W, Dhippayom T, Tan-Koi W-C, Kongkaew C (2016). Pharmacovigilance activities in ASEAN countries.. Pharmacoepidemiol Drug Saf.

[23] Olsson S, Pal SN, Dodoo A (2015). Pharmacovigilance in resource-limited countries.. Expert Rev Clin Pharmacol.

[24] Leporini C, Marrazzo G, Mumoli L, Esposito S, Gallelli L, Mangano G (2017). Adverse drug reactions reporting in Calabria (Southern Italy) in the four-year period 2011-2014: impact of a regional pharmacovigilance project in light of the new European Legislation.. Expert Opin Drug Saf.

